# MARCH8 Inhibits Ebola Virus Glycoprotein, Human Immunodeficiency Virus Type 1 Envelope Glycoprotein, and Avian Influenza Virus H5N1 Hemagglutinin Maturation

**DOI:** 10.1128/mBio.01882-20

**Published:** 2020-09-15

**Authors:** Changqing Yu, Sunan Li, Xianfeng Zhang, Ilyas Khan, Iqbal Ahmad, Yulong Zhou, Shuo Li, Jing Shi, Yu Wang, Yong-Hui Zheng

**Affiliations:** a Harbin Veterinary Research Institute, CAAS-Michigan State University Joint Laboratory of Innate Immunity, State Key Laboratory of Veterinary Biotechnology, Chinese Academy of Agricultural Sciences, Harbin, China; b National High Containment Facilities for Animal Diseases Control and Prevention, Harbin Veterinary Research Institute, Chinese Academy of Agricultural Sciences, Harbin, China; c Laboratory of Core Facility, Harbin Veterinary Research Institute, Chinese Academy of Agricultural Sciences, Harbin, China; d College of Animal Science and Technology, Heilongjiang Bayi Agricultural University, Daqing, China; e Department of Microbiology and Molecular Genetics, Michigan State University, East Lansing, Michigan, USA; Stanford University School of Medicine; Washington University School of Medicine

**Keywords:** Ebola, HIV, MARCH8, class I fusion protein, furin, glycosylation, influenza, viral envelope

## Abstract

Membrane-associated RING-CH-type 8 (MARCH8) strongly blocks human immunodeficiency virus type 1 (HIV-1) envelope glycoprotein (Env) incorporation into virions by downregulating its cell surface expression, but the mechanism is still unclear. We now report that MARCH8 also blocks the Ebola virus (EBOV) glycoprotein (GP) incorporation via surface downregulation. To understand how these viral fusion proteins are downregulated, we investigated the effects of MARCH8 on EBOV GP maturation and externalization via the conventional secretion pathway. MARCH8 interacted with EBOV GP and furin when detected by immunoprecipitation and retained the GP/furin complex in the Golgi when their location was tracked by a bimolecular fluorescence complementation (BiFC) assay. MARCH8 did not reduce the GP expression or affect the GP modification by high-mannose *N*-glycans in the endoplasmic reticulum (ER), but it inhibited the formation of complex *N*-glycans on the GP in the Golgi. Additionally, the GP *O*-glycosylation and furin-mediated proteolytic cleavage were also inhibited. Moreover, we identified a novel furin cleavage site on EBOV GP and found that only those fully glycosylated GPs were processed by furin and incorporated into virions. Furthermore, the GP shedding and secretion were all blocked by MARCH8. MARCH8 also blocked the furin-mediated cleavage of HIV-1 Env (gp160) and the highly pathogenic avian influenza virus H5N1 hemagglutinin (HA). We conclude that MARCH8 has a very broad antiviral activity by prohibiting different viral fusion proteins from glycosylation and proteolytic cleavage in the Golgi, which inhibits their transport from the Golgi to the plasma membrane and incorporation into virions.

## Abstract (precis)

Enveloped viruses express three classes of fusion proteins that are required for their entry into host cells via mediating virus and cell membrane fusion. Class I fusion proteins are produced from influenza viruses, retroviruses, Ebola viruses, and coronaviruses. They are first synthesized as a type I transmembrane polypeptide precursor that is subsequently glycosylated and oligomerized. Most of these precursors are cleaved *en route* to the plasma membrane by a cellular protease furin in the late secretory pathway, generating the trimeric N-terminal receptor-binding and C-terminal fusion subunits. Here, we show that a cellular protein, MARCH8, specifically inhibits the furin-mediated cleavage of EBOV GP, HIV-1 Env, and H5N1 HA. Further analyses uncovered that MARCH8 blocked the EBOV GP glycosylation in the Golgi and inhibited its transport from the Golgi to the plasma membrane. Thus, MARCH8 has a very broad antiviral activity by specifically inactivating different viral fusion proteins.

## INTRODUCTION

A fundamental process in cellular physiology is to externalize transmembrane proteins and soluble proteins to the plasma membrane or extracellular space after they are synthesized in the endoplasmic reticulum (ER). The vast majority of these proteins are exported by the conventional protein secretion (CPS) pathway involving the ER-to-Golgi transport, whereas the others are exported by the unconventional protein secretion pathway that is independent of the Golgi (1, 2). Proteins destined for CPS are first targeted inside the ER via signal sequences where *N*-glycosylation is initiated. *N*-Glycosylation *en bloc* transfers an oligosaccharide precursor to an Asn residue in an Asn-X-Ser/Thr (X≠Pro) motif, which includes two *N-*acetylglucosamine (GlcNAc), nine mannose, and three glucose residues. After folding and initial processing, glycoproteins with high-mannose *N*-glycans (*Nh*-glycans) exit from the ER and enter the *cis*-Golgi network (CGN), where extensive mannose trimming occurs. Within the *medial-*Golgi and the *trans*-Golgi network (TGN), complex *N*-glycans (*Nc*-glycans) and hybrid *N*-glycans (*Nhc*-glycans) are formed after adding other monosaccharides to the low-mannose precursor to complete the *N*-glycosylation process. In addition, *O*-glycosylation occurs in vertebrates by attaching *N*-acetylgalactosamine (GalNAc) to a Ser or Thr residue as a late event in the Golgi (3). Mature glycoproteins are sorted into the membranous vesicles from the TGN and externalized.

Ebola virus (EBOV), influenza A virus (IAV), and human immunodeficiency virus type 1 (HIV-1) are enveloped viruses that cause fatal hemorrhagic fever, immunodeficiency, or respiratory illnesses (4). Although they are unrelated, they express structurally similar class I fusion proteins via CPS, which consist of trimeric receptor binding, and fusogenic subunits to mediate their fusion with cell membranes (5). EBOV, HIV-1, and IAV fusion proteins are first synthesized as type I transmembrane (TM) polypeptide precursor GP_0_, gp160, or HA_0_. All these viral proteins are subjected to heavy *N*-glycosylation. *O*-Glycosylation has not been detected from IAV HA (6). Although one potential *O*-glycosylation site exists, *O*-glycans have not been found from virion-associated HIV-1 Env (7). In addition to those 27 *N*-glycosylation sites, EBOV GP has ∼80 *O*-glycosylation sites in the mucin-like domain (MLD) (8). Trimeric EBOV GP_0_, HIV-1 gp160, and HA_0_ from highly pathogenic avian influenza viruses such as H5N1 are cleaved into GP_1_/GP_2_, gp120/gp41, or HA_1_/HA_2_ heterodimers by a proprotein convertase furin in the TGN (9). These mature trimeric heterodimers are exported to the plasma membrane and incorporated into virions as spikes to initiate infection.

Membrane-associated RING-CH-type (MARCH) proteins consist of 11 members and belong to the really interesting new gene (RING)-finger E3 ligase family (10). These proteins were originally discovered from gammaherpesviruses as modulators of immune recognitions (MIRs), which promote immune evasion by downregulation of major histocompatibility complex I (MHC-I) molecules from the cell surface (11). MARCH8 is the first identified cellular MIR (c-MIR) from the human genome (12, 13), and MARCH proteins have been found to downregulate a number of cellular proteins from the cell surface (14). Recently, MARCH8 was found to block HIV-1 Env incorporation and inhibit viral replication by downregulating Env from the cell surface (15, 16). We now report that MARCH8 has a very broad antiviral activity that inhibits EBOV GP, HIV-1 Env, and H5N1 HA maturation.

## RESULTS

### MARCH8 downregulates EBOV GP from the cell surface.

We used EBOV GP to test how MARCH8 affects class I fusion protein maturation and secretion. EBOV GP has well-defined *N*- and *O*-glycosylation sites, and importantly, the GP processing and *O*-glycosylation are easily detectable after deleting the MLD region in GP_1_ that is not required for viral entry (17, 18). EBOV also expresses several different mature and soluble GP forms that are secreted or shed extracellularly, which is easily detectable by Western blotting (WB) (8). Thus, we used EBOV GP and its mutant bearing MLD deletion (ΔMLD) to study the MARCH8 activity.

**FIG 1:**
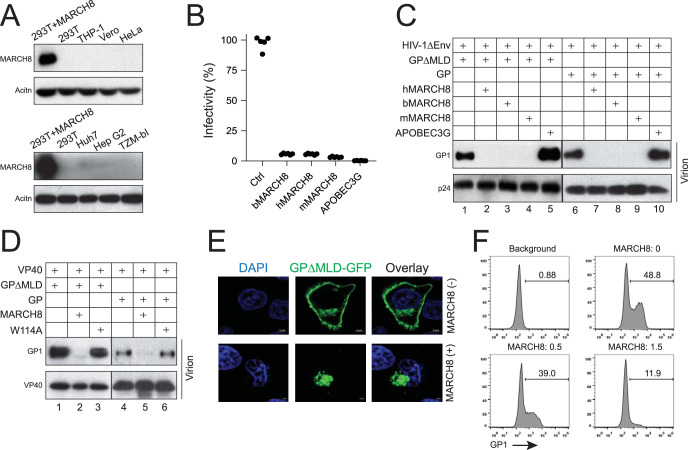
MARCH8 downregulates EBOV GP from the cell surface. (A) MARCH8 expressions in 293T, THP-1, Vero E6, HeLa, Huh7, Hep G2, and TZM-bI cells, or in 293T cells transfected with a human MARCH8 expression vector (293T+MARCH8), were analyzed by Western blotting (WB) using a specific anti-human MARCH8. (B) HIV-1 firefly luciferase-reporter pseudoviruses were produced from 293T cells in the presence of EBOV GPΔMLD and MARCH8 from different species or APOBEC3G. Viral infectivity was determined via infecting Vero E6 cells. Infectivity is shown as relative values, with the infectivity in the presence of a control (Ctrl) vector set as 100. Results were from three different experiments. h, human; b, cow (Bos taurus); m, mouse. (C) HIV-1 pseudoviruses were collected from cell culture supernatants in panel B and purified via ultracentrifugation. In addition, HIV-1 pseudoviruses containing the full-length EBOV GP were produced and purified similarly. The levels of GP_1_ expression in these VLPs were analyzed by WB using anti-FLAG, and the levels of VLPs were determined by anti-HIV-1 Gag (p24). Protein expressions in 293T cells are shown in Fig. 4D. (D) EBOV virus-like particles (VLPs) were produced from 293T cells after expressing EBOV VP40, human MARCH8 or its W114A mutant, and EBOV GP or GPΔMLD. After purification via ultracentrifugation, these VLPs were analyzed by WB. Protein expressions in viral producer cells are shown in Fig. 4C. (E) EBOV GPΔMLD that has a GFP tag was expressed in HeLa cells in the presence or absence of human MARCH8. After staining with DAPI, the GP localization was detected by confocal microscopy. (F) EBOV GPΔMLD was expressed with increasing amounts of human MARCH8 in 293T cells. The GP expression on cell surface was detected by flow cytometry using anti-FLAG followed by Alexa Fluor 488-conjugated goat anti-mouse IgG.

MARCH8 is expressed in terminally differentiated myeloid cells (15). We could not detect MARCH8 expression in commonly used human cell lines including 293T, Vero, THP1, Huh7, Hep G2, HeLa, and HeLa-derived TZM-bI cells (Fig. 1A). Thus, we investigated the MARCH8 antiviral activity via ectopic expression. Initially, we tested how MARCH8 proteins from human (h), cow (Bos taurus, b), and mouse (m) affect EBOV entry using pseudotyped HIV-1, which is a faithful system for studying the EBOV GP function (18–21). All these MARCH8 proteins restricted the pseudotyped virus replication as effectively as the retroviral restriction factor APOBEC3G (Fig. 1B). To explore the MARCH8 antiviral mechanism, EBOV virus-like particles (VLPs) and HIV-1 VLPs were produced by expressing EBOV VP40 or HIV-1 Gag proteins in the presence of MARCH8 and GP or GPΔMLD. MARCH8 strongly reduced the GP incorporation into these virions (Fig. 1C and D). In contrast, the inactive RING domain mutant W114A did not have such activity (Fig. 1D). When GP subcellular localization was determined by confocal microscopy, although GP alone was detected predominantly on the plasma membrane, it was retargeted into the cytoplasm when MARCH8 was expressed (Fig. 1E). Consistently, GP was strongly downregulated from the cell surface by MARCH8 in a dose-dependent manner when detected by flow cytometry (Fig. 1F).

### MARCH8 retains EBOV GP in the Golgi.

**FIG 2:**
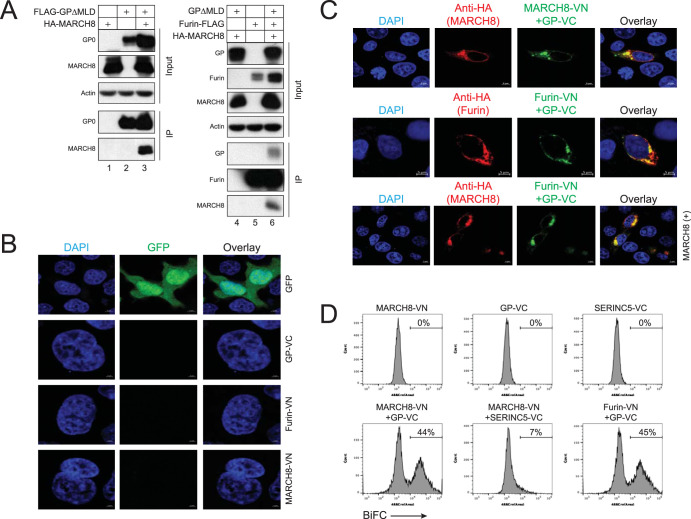
Tracking MARCH8, EBOV GP, and furin protein complexes by BiFC. (A) FLAG-tagged GPΔMLD was expressed with HA-tagged human MARCH8. In addition, FLAG-tagged furin was expressed with HA-tagged human MARCH8 and GPΔMLD in 293T cells. Proteins were immunoprecipitated with anti-FLAG and analyzed by WB. MARCH8, furin, and GP were detected by anti-HA, anti-FLAG, or anti-EBOV GP, respectively. (B) GFP, GP-VC, furin-VN, or human MARCH8-VN proteins were individually expressed in HeLa cells. After staining with DAPI, fluorescent signals were observed by confocal microscopy. (C) The MARCH8-VN-HA/GP-VC or furin-VN-HA/GP-VC BiFC pair was expressed in HeLa cells. Cells were stained with fluorescent anti-HA to detect MARCH8 or furin. In addition, furin-VN that does not express the HA tag was expressed with GP-VC and HA-tagged MARCH8 in HeLa cells. Cells were stained with fluorescent anti-HA to detect MARCH8. Fluorescent signals were observed by confocal microscopy. (D) Indicated BiFC fusion proteins were expressed individually or pairwise, and the levels of BiFC signals were determined by flow cytometry. SERINC5, serine incorporator 5. Experiments were repeated three times, and identical results were obtained.

To understand where GP is targeted by MARCH8, we studied GP and MARCH8 subcellular localization. First, we tested their interactions by immunoprecipitation. GPΔMLD could specifically pull down MARCH8 (Fig. 2A, lanes 1 to 3), and furin could pull down GPΔMLD and MARCH8 (Fig. 2A, lanes 4 to 6). These results demonstrate that GP, furin, and MARCH8 interact with each other in cells.

Second, we set up a bimolecular fluorescence complementation (BiFC) assay to track their interactions in live cells (22). The C termini of GP, MARCH8, and furin were fused to the N-terminal 2 to 173 amino acids (aa) of a green fluorescent protein Venus (VN) or its C-terminal 154 to 238 aa (VC). As a control, serine incorporator 5 (SERINC5) protein was also fused to VC. When GP-VC, furin-VN, and MARCH-VN were expressed individually, no green BiFC signal was detected by confocal microscopy (Fig. 2B). When GP-VC was expressed with MARCH8-VN or furin-VN, strong green fluorescent signals were produced that overlapped MARCH8 or furin (Fig. 2C), confirming the interactions between GP and MARCH8, or GP and furin. Unlike GP alone that was found on the plasma membrane (Fig. 1E), the GP-MARCH8 BiFC complex was found in intracellular compartments, confirming that MARCH8 downregulates GP from the cell surface. In addition, when the furin-VN/GP-VC pair was expressed with MARCH8, their colocalization was also detected (Fig. 2C), confirming that these three molecules form a complex in live cells. When these interactions were quantified by flow cytometry, both MARCH8/GP and furin/GP pairs generated ∼45% BiFC-positive cells, whereas positive cells produced from the MARCH8/SERINC5 pair were only around 7% (Fig. 2D). Thus, we detected specific MARCH8-GP and furin-GP interactions via BiFC.

**FIG 3:**
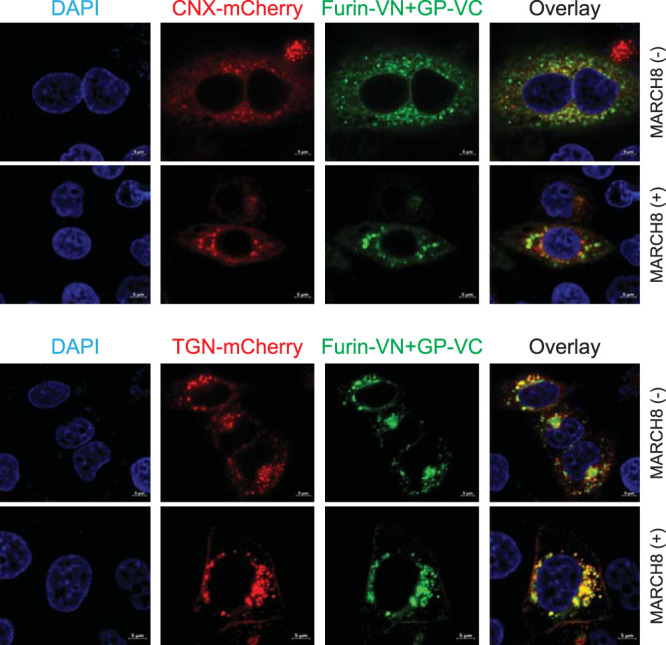
MARCH8 retains EBOV GP in the Golgi. The furin-VN/GP-VC BiFC pair was expressed with CNX-mCherry or TGN-mCherry in the presence or absence of human MARCH8 in HeLa cells. After staining with DAPI, fluorescent signals were observed by confocal microscopy.

Third, we determined how MARCH8 affects the GP and furin subcellular localization via confocal microscopy. The furin-VN/GP-VC BiFC pair was expressed with an ER marker, calnexin (CNX), or a TGN marker that was fused with mCherry. Although the GP-furin complex colocalized with both CNX and TGN, its colocalization with TGN was much stronger than that with CNX when MARCH8 was not expressed (Fig. 3). In the presence of MARCH8, its colocalization with TGN was strongly increased, whereas that with CNX was decreased. These results suggest that MARCH8 retains EBOV GP in the Golgi.

### MARCH8 blocks EBOV GP proteolytic processing.

**FIG 4:**
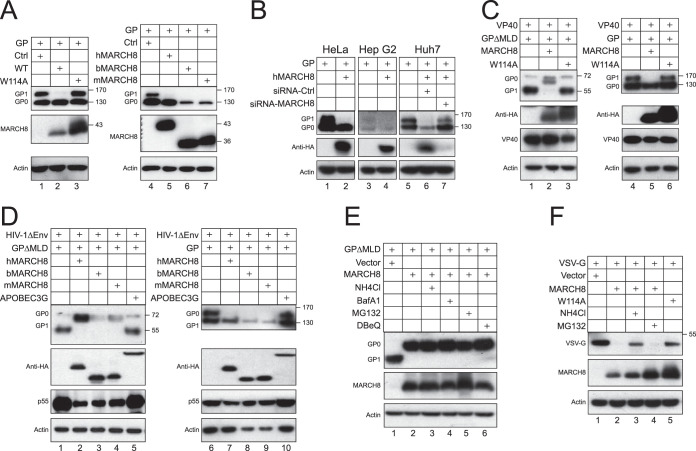
MARCH8 blocks the EBOV GP proteolytic cleavage. (A) GP was expressed with wild-type (WT) human MARCH8 or its W114A mutant in 293T cells. The GP and MARCH8 expression was detected by WB using anti-FLAG or anti-HA. Activities of MARCH8 proteins from different species were also compared. (B) GP was expressed with human MARCH8 in HeLa and Hep G2 cells, or in Huh7 cells in the presence of control (Ctrl) or MARCH8-specific siRNAs. The GP processing was determined by WB. (C) Similar experiments as in panel A were conducted to determine how MARCH8 and its W114A mutant affect GP cleavage in the presence of EBOV VP40. (D) Similar experiments as in panel A were conducted to determine how MARCH8 proteins from different species affect GP cleavage in the presence of HIV-1 proteins. (E) GPΔMLD was expressed with human MARCH8 in 293T cells and treated with NH_4_Cl, bafilomycin A1 (BafA1), MG132, or DBeQ. Protein expressions were determined by WB. (F) VSV-G was expressed with human MARCH8 or its W114A mutant in 293T cells and treated with NH_4_Cl or MG132. Protein expressions were determined by WB. Numbers beside gels in this and subsequent figures indicate molecular masses in kilodaltons.

The full-length GP was expressed with MARCH8 from different species or its W114A mutant, and GP processing was analyzed by comparing GP_0_ and GP_1_ expression levels via WB. As reported previously (18), because of the heavy *O*- and *N*-glycosylation of MLD in the Golgi, GP_1_ exhibited a higher molecular weight than GP_0_ (Fig. 4A). When MARCH8 proteins were expressed, GP_1_ proteins were almost undetectable, indicating that MARCH8 blocks the GP processing. The W114A mutant was inactive, indicating that the RING domain is required for this activity. To understand whether this activity is cell type specific, similar experiments were conducted in HeLa, Hep G2, and Huh7 cells. Again, the GP_1_ expression in these cells was selectively inhibited by MARCH8 (Fig. 4B). To confirm the specificity of this MARCH8 effect, Huh7 cells were also transfected with small interfering RNAs (siRNAs) to silence the MARCH8 expression. When the MARCH8 expression was decreased, the GP_1_ expression was effectively recovered (Fig. 4B, lanes 5 to 7). Thus, the MARCH8 effect is not cell type specific.

To understand whether this MARCH8 activity was affected by the mucin-like domain, the full-length GP and GPΔMLD processing were compared side-by-side in the presence of wild-type (WT) MARCH8 or its W114A mutant (Fig. 4C), or MARCH8 proteins from different species (Fig. 4D). Unlike GP_1_ from the full-length GP, GP_1_ΔMLD exhibited a lower molecular weight than GP_0_ΔMLD. In addition, unlike similar levels of GP_0_ and GP_1_ from the full-length GP, the GP_1_ levels were much higher than GP_0_ from GPΔMLD, confirming that GPΔMLD is more effectively processed than the full-length GP. Nevertheless, GP_1_ expressions from both full-length GP and GPΔMLD were completely inhibited by MARCH8 proteins but not by the W114A mutant (Fig. 4C and D).

To test whether MARCH8 destabilizes GP_1_, cells were treated with inhibitors that target different degradation pathways, including MG132 for proteasomes, NH_4_Cl and bafilomycin A1 (BafA1) for lysosomes, and DBeQ for ER-associated protein degradation (ERAD). None of these inhibitors rescued GP_1_ expression or increased GP_0_ expression, indicating that GP_1_ is not subjected to degradation by these two pathways (Fig. 4E).

We also tested how MARCH8 affects the expression of vesicular stomatitis virus glycoprotein (VSV-G), a class III fusion protein. The VSV-G expression was strongly reduced by MARCH8 but not by its W114A mutant, and the reduction was blocked by NH_4_Cl but not MG132 (Fig. 4F), confirming that MARCH8 triggers VSV-G degradation via the lysosome pathway as reported previously (15).

**FIG 5:**
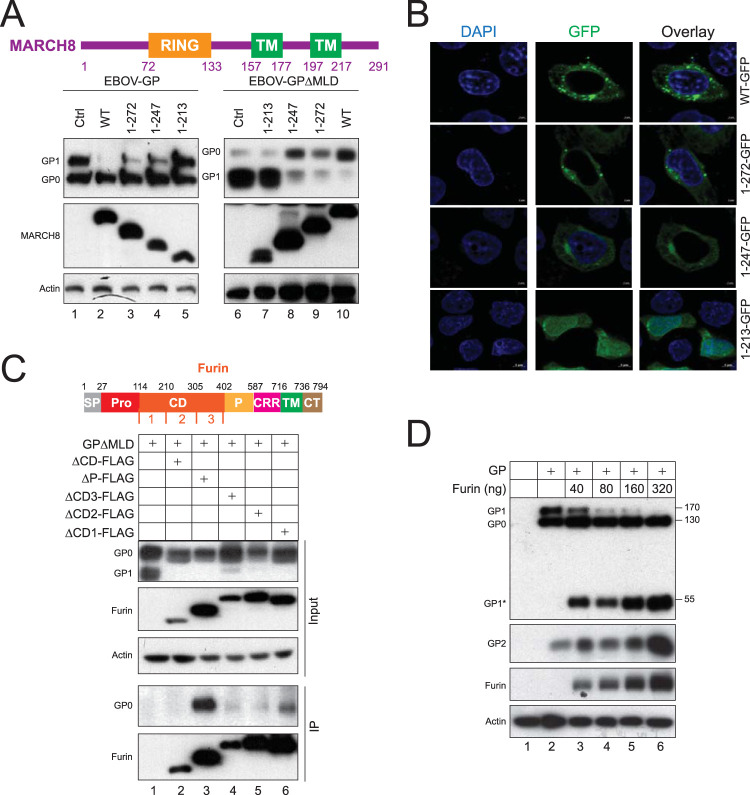
Mapping MARCH8 and furin active domains. (A) A schematic diagram of human MARCH8 protein with a RING domain and two TM domains is presented. Three MARCH8 C-terminal CT deletion mutants, 1–213, 1–247, and 1–272, were created and expressed with the full-length GP or GPΔMLD. MARCH8 and GP expression was detected by WB using anti-HA or anti-FLAG. (B) MARCH8 and its three deletion mutants that have a C-terminal GFP tag were expressed in HeLa cells. After staining with DAPI, their subcellular localizations were detected by confocal microscopy. (C) A schematic diagram of human furin functional domains is presented. FLAG-tagged CD, P domain, and CD subdomain-deletion mutants were created and were expressed with GPΔMLD. Proteins were immunoprecipitated by anti-FLAG and detected by WB. Furin was detected by anti-FLAG, and EBOV GP was detected by anti-EBOV GP antibodies. (D) The full-length GP was expressed with increasing amounts of furin in 293T cells. The GP and furin expression was determined by WB.

Human MARCH8 has 291 amino acids (aa) that create a RING-CH finger in the N-terminal cytoplasmic tail (CT), two TM domains, and a C-terminal CT (Fig. 5A). The critical W114 residue is in the RING region and is required for recruitment of ubiquitin-conjugating E2 enzyme (23). To uncover other important domains, we created three MARCH8 C-terminal CT deletion mutants by expressing its 1-to-213-aa (1–213), 1-to-247-aa (1–247), and 1-to-272-aa (1–272) regions. When these mutants were expressed with the full-length GP or GPΔMLD, WT and mutants 1–247 and 1–272 blocked the GP processing, whereas the 1–213 mutant did not (Fig. 5A). These mutants were then fused with a green fluorescent protein (GFP) tag, and their subcellular localizations were tracked and compared by confocal microscopy. Both WT and the 1–272 mutant displayed a scattered punctate localization in the cytoplasm, whereas the 1–213 mutant exhibited diffuse localization in the cytoplasm and nucleus (Fig. 5B). The 1–247 mutant diffused partially in the cytoplasm. These results demonstrate that the 213–247 region determines MARCH8 intracellular compartmentalization that is critical for its activity.

### Identification of a novel furin cleavage site on EBOV GP.

Human furin is a 794-aa type I TM protein with a large lumenal region that has a signal peptide (SP), prodomain (Pro), subtilisin-like catalytic domain (CD), P domain, cysteine-rich region (CRR), TM domain, and a 56-aa CT (24) (Fig. 5C). The CD and P domain are functionally critical for furin convertase activities. We created five furin dominant negative mutants, including ΔCD, ΔP, ΔCD1, ΔCD2, and ΔCD3, by deleting CD (114 to 402 aa), P domain (402 to 587 aa), and the three CD subdomains CD1 (114 to 210 aa), CD2 (210 to 305 aa), and CD3 (305 to 402 aa). When these five mutants were expressed with GP, they all blocked the GP cleavage from GP_0_ to GP_1_ (Fig. 5C, lanes 2 to 6), confirming that EBOV GP is processed by furin (25). Consistently, when their interactions were determined by immunoprecipitation, GP was pulled down by the ΔP mutant, but not by the ΔCD, ΔCD1, ΔCD2, and ΔCD3 mutants (Fig. 5C), indicating that GP interacts with the furin catalytic domain.

To detect furin-cleaved EBOV GP products, full-length GP was expressed with increasing amounts of furin. GP_1_ and GP_2_ were detected even in the absence of ectopic furin, which is produced by endogenous furin (Fig. 5D, lane 2). In the presence of ectopic furin, although the levels of GP_0_ were not changed, those of GP_1_ and GP_2_ were decreased or increased in a dose-dependent manner by furin (Fig. 5D, lanes 3 to 6). In addition, there appeared another GP at ∼55 kDa that was also increased by furin. Accordingly, a novel furin cleavage site, ^299^RKIR^302^, was found in GP_1_ in front of MLD, and this novel 55-kDa product was named GP_1_* (see Fig. 7B and also Fig. S1 in the supplemental material). We conclude that GP_1_* is predominantly processed from GP_1_ by furin.

10.1128/mBio.01882-20.1 FIG S1 Identification of a novel furin cleavage site in EBOV GP_1_. Two furin cleavage sites in EBOV GP are indicated in the sequence by red, including the novel ^299^RKIR^302^ site that produces GP_1_*. Download FIG S1, PDF file, 0.4 MB. Copyright © 2020 Yu et al. 2020 Yu et al. This content is distributed under the terms of the Creative Commons Attribution 4.0 International license.

### MARCH8 blocks EBOV GP modification by *Nc*- and *O*-glycans.

**FIG 6:**
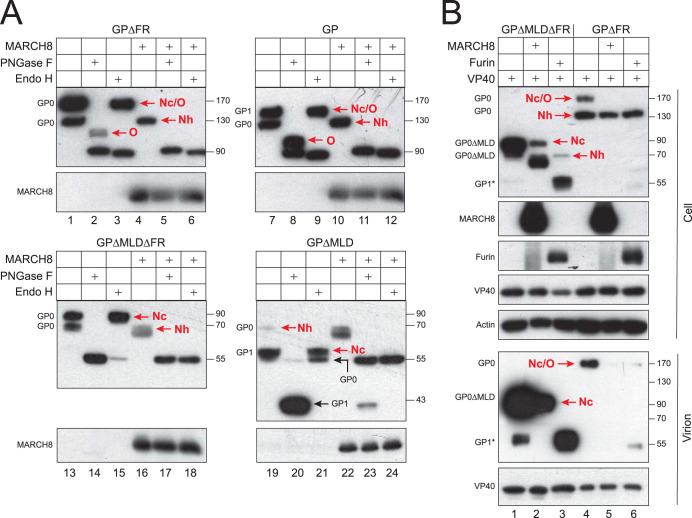
MARCH8 blocks the EBOV GP modification by *Nc*- and *O*-glycans. (A) GP, GPΔFR, GPΔMLD, or GPΔMLDΔFR was expressed with human MARCH8 in 293T cells. After lysis, cell lysate was treated with Endo H or PNGase F, or left untreated, and analyzed by WB. (B) GPΔFR and GPΔMLDΔFR were expressed with EBOV VP40 and human MARCH8 or furin in 293T cells. EBOV VLPs were purified from supernatants via ultracentrifugation. EBOV GP, MARCH8, or furin expressions in cells and/or VLPs were determined by WB.

Analysis of the GP glycosylation becomes complex due to proteolytic processing. To detect GP glycosylation, we blocked GP processing by removal of the furin cleavage site between GP_1_ and GP_2_. Two furin cleavage site-deficient (ΔFR) mutants were created from the full-length GP and GPΔMLD, and GP glycosylation was analyzed by treatments with endoglycosidase H (Endo H) and peptide-*N*-glycosidase F (PNGase F) that trim off *Nh*-glycans or all *N*-glycans, respectively. GPΔFR was detected at 170 and 130 kDa, and GPΔMLDΔFR was detected at 90 and 70 kDa (Fig. 6A, lanes 1 and 13). All these proteins were sensitive to PNGase F, confirming that they are modified by *N*-glycans (Fig. 6A, lanes 2 and 14). A ∼110-kDa, PNGase F-resistant protein was detected from GPΔFR after treatment with PNGase, confirming that GP but not GPΔMLD is modified by *O*-glycans (Fig. 6A, lane 2). The 170-kDa GPΔFR and 90-kDa GPΔMLDΔFR were resistant to Endo H (Fig. 6A, lanes 3 and 15), indicating that they are modified by *Nc*-glycans. The 130-kDa GPΔFR and 70-kDa GPΔMLDΔFR were sensitive to Endo H (Fig. 6A, lanes 3 and 15), indicating that they are modified by *Nh*-glycans. When MARCH8 was expressed, only the Endo H-sensitive 130-kDa GPΔFR and 70-kDa GPΔMLDΔFR were detected (Fig. 6A, lanes 4 to 6 and lanes 16 to 18).

Additional experiments were conducted to verify these results by using the full-length GP and GPΔMLD that have an active furin cleavage site. Much more *O*-glycosylated GP proteins were detected from the full-length GP, which should come from GP_1_ (Fig. 6A, lane 8). All GP_0_ proteins were sensitive to, whereas all GP_1_ proteins were resistant to, Endo H (Fig. 6A, lanes 9 and 21), confirming that GP_0_ and GP_1_ are modified by *Nh*- or *Nc*-glycans, respectively. Again, MARCH8 selectively inhibited the Endo-H-resistant and PNGase F-resistant GP expressions (Fig. 6A, lanes 10 to 12 and lanes 22 to 24). Collectively, these results demonstrate that MARCH8 blocks GP modifications by *Nc*- and *O*-glycans, but not by *Nh*-glycans.

### Modification of EBOV GP by *Nc*- and *O*-glycans is required for its proteolytic cleavage by furin and incorporation into virions.

Because GP_0_ is packaged into EBOV virions (26), we addressed how glycosylation affects its incorporation into EBOV VLPs. GPΔFR and GPΔMLDΔFR were expressed with EBOV VP40 and MARCH8, and their expressions in VLPs were compared. We confirm again that MARCH8 selectively inhibited the expression of GP_0_ with *Nc*- and *O*-glycans (Fig. 6B, lanes 1, 2, 4, and 5). Notably, only GP_0_ with these two types of glycans was detected from VLPs (Fig. 6B), indicating that only GP_0_ proteins with *Nc*- and *O*-glycans are selectively incorporated into virions.

When MARCH8 was replaced with furin in this experiment, the expression of GP_0_ with *Nc*- and *O*-glycans was also inhibited, but GP_1_* showed up that was also incorporated into virions (Fig. 6B, lanes 3 and 6). GP_1_* was produced much more from GPΔMLDΔFR than GPΔFR, and it was also detected from GPΔMLDΔFR in the absence of ectopic furin expression that was also enriched in virions (Fig. 6B, lane 1). Collectively, these results demonstrate that furin selectively cleaves GP_0_ with *Nc*- and *O*-glycans. In addition, because GP_1_* is incorporated into VLPs (Fig. 6B, lanes 1, 3, and 6), we confirm that only fully glycosylated GPs are incorporated into virions. Because GP_1_ is modified by *Nc*- and *O*-glycans, it further confirms that furin selectively processes fully glycosylated GP.

### MARCH8 blocks EBOV GP shedding and secretion.

**FIG 7:**
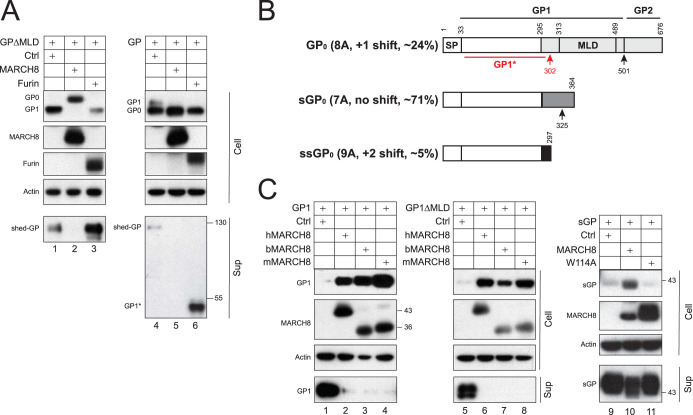
MARCH8 blocks the EBOV GP shedding and secretion. (A) Full-length GP and GPΔMLD that have an N-terminal FLAG tag were expressed with MARCH8 or furin. GP proteins released into supernatants were immunoprecipitated using anti-FLAG. The GP and MARCH8 expression in cells or supernatants was detected by WB. (B) A schematic diagram for EBOV GP, secreted GP (sGP), and small secreted GP (ssGP) expression is presented. Their relative expression levels from nonedited (7A) and edited (8A, 9A) GP mRNAs and a novel furin cleavage site in GP_1_ are shown. (C) GP_1_, GP_1_ΔMLD, and sGP that have an N-terminal FLAG tag were expressed with indicated MARCH8 proteins in 293T cells. GP proteins released into supernatants were immunoprecipitated as in panel A. The GP and MARCH8 protein expression in cells and supernatants was detected by WB. Sup, supernatants.

After maturation, GP_1_/GP_2_ trimers on the plasma membrane are further cleaved by the tumor necrosis factor alpha (TNF-α) converting enzyme (TACE) at the GP_2_ membrane-proximal external region to release a soluble shed GP (27). To verify MARCH8’s inhibitory effect on EBOV GP maturation, we tested how MARCH8 affects GP shedding. After expression of GP and GPΔMLD with MARCH8 or furin, GP proteins were immunoprecipitated from supernatants and analyzed by WB. MARCH8 strongly blocked GP shedding, and in contrast, furin strongly increased it (Fig. 7A). GP_1_* was also detected as soluble proteins.

In addition to GP, EBOV produces secreted GP (sGP) and small secreted GP (ssGP) via RNA editing, all of which share the N-terminal 295 aa (Fig. 7B). To address how MARCH8 affects the GP secretion, we expressed sGP with MARCH8 proteins and measured its secretion. Unlike its W114A mutant, MARCH8 retained sGP in cells and effectively reduced its secretion (Fig. 7C, lanes 9 to 11). Next, we expressed only GP_1_ or GP_1_ΔMLD that should also be secreted as sGP and tested how MARCH8 affects their secretion. MARCH8 proteins from different species strongly increased GP_1_ and GP_1_ΔMLD expression in cells but completely blocked their secretions (Fig. 7C, lanes 1 to 8).

### MARCH8 blocks HIV-1 Env and H5N1 HA maturation.

Unlike HIV-1 Env, most IAV HAs are processed by trypsin-like serine proteases. However, a highly pathogenic avian strain such as H5N1 has gained an additional furin cleavage site to expand its tropism (28). Thus, we used H5N1 HA as well as HIV-1 Env to confirm the MARCH8 antiviral activity.

**FIG 8:**
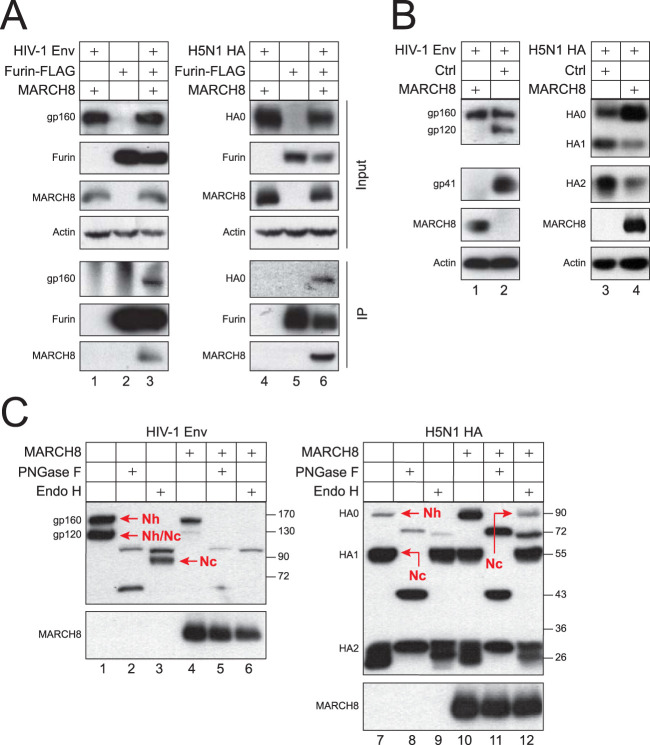
MARCH8 blocks the HIV-1 Env and H5N1 HA maturation. (A) FLAG-tagged furin was expressed with MARCH8 and HIV-1 Env or H5N1 HA in 293T cells. Proteins were immunoprecipitated with anti-FLAG and analyzed by WB. (B) HIV-1 Env and H5N1 HA were expressed with MARCH8 in 293T cells, and their processing was determined by WB. (C) HIV-1 Env and H5N1 HA were expressed with MARCH8 in 293T cells. After lysis of cells, cell lysate was treated with Endo H or PNGase F, or left untreated, and analyzed by WB.

When HIV-1 Env and H5N1 HA were expressed with furin and MARCH8, furin pulled down gp160 or HA_0_ with MARCH8 (Fig. 8A), confirming that MARCH8 interacts with Env/furin or HA/furin complex. In addition, when MARCH8 was expressed with these fusion proteins, HIV-1 gp120/gp41 and H5N1 HA_1_/HA_2_ expression levels were all decreased (Fig. 8B), confirming that MARCH8 also blocks the Env and HA cleavage by furin.

When the Env and HA glycosylations were analyzed after treatments with glycosidases, they were all sensitive to PNGase F (Fig. 8C, lanes 2 and 8), confirming that they are modified by *N*-glycans. HIV-1 gp160 was completely sensitive to Endo H, indicating that it is modified by *Nh*-glycans (Fig. 8C, lane 3). HIV-1 gp120 was only partially sensitive to Endo H (Fig. 8C, lane 3), indicating that it is modified by both *Nh*- and *Nc*-glycans. H5N1 HA_0_ and HA_1_ were completely sensitive or resistant to Endo H (Fig. 8C, lane 9), indicating that HA_0_ and HA_1_ were modified by *Nh*- or *Nc*-glycans, respectively. When MARCH8 was expressed, the gp120 expression was reduced likely due to inhibition of the furin activity (Fig. 8C, lane 4). In addition, Endo H-sensitive HA_0_ expression was increased (Fig. 8C, lane 12). Collectively, these results demonstrate that MARCH8 does not block the Env and HA modification by *Nh*-glycans in the ER, but rather they suggest that it should target the other glycosylation steps.

## DISCUSSION

Class I fusion proteins project from the virion surface as highly glycosylated heterotrimeric spikes and initiate infection by binding to receptors on the surface of target cells. Although it is clear that glycosylation and proteolytic cleavage are critically important, it remains incompletely understood how these steps are coopted to generate mature spikes that are incorporated from the plasma membrane into virions. We now show that MARCH8 specifically inhibits these processes, which provides new understanding for this poorly defined mechanism.

Our results highlight the fundamental role of *N*-glycosylation in the spike formation. First, *N*-glycosylation is absolutely required for EBOV GP maturation. Although MARCH8 does not affect the GP modification by *Nh*-glycans in the ER, it inhibits the GP modification by *Nc*- and *O*-glycans as well as its proteolytic processing in the TGN. These results suggest that MARCH8 inhibits the conversion of *Nh*-glycans to *Nc*-glycans, likely by blocking the mannose trimming step in the CGN and/or the following glycosylation steps in the *medial*-Golgi and the TGN. In addition, it is possible that *O*-glycosylation and furin cleavage are both dependent on the mannose trimming. In fact, when GPΔFR and GPΔMLDΔFR were expressed with furin, only GP_0_ proteins with *Nc*- and *O*-glycans were processed into GP_1_* (Fig. 6B, lanes 3 and 6). Consistently, when GP was expressed with furin, only GP_1_ but not GP_0_ was effectively processed into GP_1_* (Fig. 5D). These results strongly suggest that the GP processing by furin is dependent on the completion of the glycosylation processes. Second, *N*-glycosylation is required for EBOV GP transport from the TGN to the plasma membrane. MARCH8 not only blocked GP shedding and secretion (Fig. 7), but also downregulated GP expression on the cell surface (Fig. 1E and F). These inhibitory effects were likely caused by an inhibition of the GP glycosylation in the Golgi by MARCH8. In fact, we found that only GP proteins modified with *O*- and/or *Nc*-glycans were selectively incorporated into virions (Fig. 6B). Thus, glycosylation is required for GP externalization and incorporation into virions. Because we detected the GP interaction with MARCH8 as well as the GP retention in the TGN by MARCH8, it is possible that MARCH8 directly or indirectly retains viral glycoproteins at an early location so they never physically reach the location where they would be glycosylated. In that case all the glycosylation machinery would be intact but the GP would never reach it.

In addition to the three class I fusion proteins, MARCH8 also targets a class III fusion protein, VSV-G, via a different mechanism. Instead of inhibiting maturation, MARCH8 triggers VSV-G degradation via the lysosomal pathway. Although MARCH8 does not trigger class I fusion protein degradation, its activity still depends on the RING domain, because the W114A mutant that has a dead RING domain is inactive. These results demonstrate that the MARCH8 activity depends on the ubiquitination pathway. We speculate that MARCH8 might ubiquitinate and degrade a critical cellular transport facilitator, which blocks GP from access to the glycosylation and furin cleavage machinery. However, the degradation of VSV-G by MARCH8 follows a similar mechanism by which MARCH proteins downregulate immune receptors from the cell surface (29). MARCH proteins usually ubiquitinate a lysine residue in the cytoplasmic tail of these immune receptors, which redirects them from the endosomes to the late endosomes and the lysosomes for degradation when they are endocytosed from the cell surface (14). Thus, MARCH proteins use two different mechanisms to target different proteins. For class I fusion proteins, MARCH8 blocks their anterograde transport from the TGN to the plasma membrane; for VSV-G and immune receptors, MARCH proteins block their recycling back to the TGN after retrograde transport from the plasma membrane to the endosomes.

In addition to these four viral proteins, MARCH proteins can target an amazingly large number of cellular proteins (14). Most of these targets are immune receptors on the cell surface that play an important role in innate and adaptive immunity, including MHC I, MHC II, CD4, CD44, CD81, CD86, CD95, CD98, intracellular adhesion molecule (ICAM)-1, NKG2D ligand Mult1, transferrin receptor (TfR), TRAIL receptor 1, and interleukin-1 receptor (IL1RAP). Thus, the expression of MARCH proteins is tightly regulated, which causes their poor expression in immortalized cell lines and a major difficulty in studying their endogenous function (10). Thus, we were unable to further confirm the MARCH8 antiviral activity by silencing its endogenous gene expression. In addition, due to lack of structural data on MARCH proteins, it is difficult to understand how they have so many different targets. The role of TM domains of MARCH proteins in substrate recognition has been suggested (14). A GxxxG motif is conserved in MARCH TM domains, and it is also present in the MHC II TM domain, which may mediate their interactions. In addition, the recognition could be mediated by a common adaptor protein.

In addition to MARCH8, the maturation of viral fusion proteins is inhibited by guanylate-binding proteins (GBP) 2 and 5 (30), and interferon-induced transmembrane (IFITM) protein 3 (31). GBP2/5 directly target the cytoplasmic part of furin and inhibit its enzymatic activities, which exert broad antiviral activity by inhibiting furin-mediated cleavage of viral fusion proteins. IFITM3 interrupts viral glycoprotein processing of murine leukemia virus (MLV) Env, HIV-1 Env, and VSV-G, but not EBOV GP, suggesting that it does not affect furin function. In addition, it interferes with Env trafficking and promotes its degradation in lysosomes. Thus, mammalian cells have evolved diverse mechanisms to intercept the production of mature viral fusion proteins for assembly of infectious virus particles.

MARCH8 is naturally expressed in terminally differentiated myeloid cells such as macrophages and dendritic cells (DCs). These cells are the primary targets for HIV-1 and EBOV infection, transmission, and dissemination, and establishment of persistent tissue virus reservoirs in the case of HIV-1. These cells can also be infected by H5N1, although the infection may not necessarily contribute to the significant part of pathogenesis. Thus, MARCH8 should play a role in defending these viruses during natural infection. In fact, when the MARCH8 expression was silenced in macrophages, HIV-1 replication was strongly promoted (15). Although macrophages and DCs express all HIV-1 receptors, they are much less permissive to HIV-1 infection than CD4^+^ T lymphocytes. The HIV-1 resistance in these myeloid cells is currently attributed to their high expression levels of the host restriction factor SAMHD1. However, the role of MARCH8 should not be ignored. In addition, it should be addressed how viruses counteract MARCH8 to establish productive infection.

## MATERIALS AND METHODS

### Cell lines, RNA oligonucleotides, and transfection reagents.

HEK293T, HeLa, Hep G2, Huh7, Vero E6, and TZM-bI cells were cultured in Dulbecco’s modified Eagle medium (DMEM). THP-1 cells were cultured in Roswell Park Memorial Institute (RPMI) 1640 medium. Media were supplemented with 10% fetal bovine serum and 1% penicillin-streptomycin (Gibco), and cells were cultured at 37°C in a humidified atmosphere in a 5% CO_2_ incubator. MARCH8 sense/antisense RNA oligonucleotides 5′-CCUUCUCUCGCACUUCUAUTT-3′/5′-AUAGAAGUGCGAGAGAAGGTT-3′ and negative-control sense/antisense RNA oligonucleotides 5′-UUCUCCGAACGUGUCACGUTT-3′/5′-ACGUGACACGUUCGGAGAATT-3′ were ordered from GenePharma. 293T cells were transfected with polyethylenimine (PEI) from Sigma, Huh7 cells were transfected with Lipofectamine 3000 from Thermo Fisher, and Hep G2 cells were transfected by Amaxa Nucleofector using program T-028 from Lonza.

### Plasmids.

The human MARCH8 expression plasmid pCAGGS-3xHA-hMARCH8 and its W114 mutant pCAGGS-3xHA-hMARCH8-W114A were provided by Kenzo Tokunaga (15). Bovine and murine MARCH8 cDNAs were amplified from bovine peripheral blood mononuclear cells or mouse RAW 264.7 cells by RT-PCR and cloned into pCAGGS with a C-terminal HA tag after EcoRI/XhoI digestion. The human MARCH8 C-terminal deletion mutant 1–272, 1–247, and 1–213 expression vectors were created by PCR in pCAGGS-3xHA-hMARCH8 vector. EBOV GP and EBOV VP40 expression vectors were described previously (18). The N-terminal FLAG-tagged EBOV GPΔMLD expression vector was provided by Shan-Lv Liu. pcDNA3.1-FLAG-sGP, pcDNA3.1-FLAG-GP_1_, and pcDNA3.1-FLAG-GP_1_ΔMLD were created by PCR from the EBOV-GP and EBOV GPΔMLD vector and cloned into pcDNA3.1(+) vector by BamHI/EcoRI digestion. pCMV6-Furin-FLAG/MYC was purchased from OriGene (catalogue number RC204279). The furin ΔSD, ΔSD1, ΔSD2, ΔSD3, and ΔP mutants were created in the same furin expression vector by overlapping PCR. pcDNA3.1-hMARCH8-GFP and pcDNA3.1-GPΔMLD-GFP were constructed by inserting MARCH8 and EBOV GPΔMLD cDNAs into pEGFP-N1 vector (GenBank accession no. U55762) via EcoRI/AgeI digestion.

To construct BiFC expression vectors, the furin, MARCH8, and GP-ΔMLD cDNAs were inserted into pcDNA3.1-VN, pcDNA3.1-VN-HA, or pcDNA3.1-FLAG-VC (C-terminal VN/VC) vectors by EcoRI/AgeI digestion, which generated pcDNA3.1-Furin-VN, pcDNA3.1-Furin-VN-HA, pcDNA3.1-MARCH8-VN-HA, or pcDNA3.1-GPΔMLD-FLAG-VC, respectively. pcDNA3.1-SERINC5-FLAG-VC was reported previously (32). mCherry-TGNP-N-10 and mCherry-Calnexin-N-14 expression vectors were obtained from Michael Davidson via Addgene. H5N1 HA expression vector 7705 was obtained from Gary Nabel.

### Confocal microscopy assay.

A total of 2 × 10^5^ HeLa cells were seeded on a glass slide 10 h before transfection. Cells were transfected with Lipofectamine 3000 reagent (Thermo Fisher). After 36 h, cells were washed and fixed with 4% paraformaldehyde and permeabilized with 0.1% Triton X-100. After washing, cells were blocked with 5% bovine serum albumin. For immunofluorescence assay, cells were incubated with anti-HA primary antibody for 1 h at room temperature (RT). After washing 3 times with phosphate-buffered saline (PBS), cells were stained with Alexa Fluor-647 conjugated secondary antibody 1 h at RT. Following washing 3 times with PBS, cells were stained with 4′,6-diamidino-2-phenylindole (DAPI) for 1 min, washed for 2 h at RT, and observed by a confocal microscopy assay (Zeiss LSM880). At least 100 random cells per slide were analyzed, and the most representative images from each slide were selected for presentation.

### Flow cytometry analysis.

To detect EBOV GP expression on the cell surface, a total of 2 × 10^5^ 293T were seeded in each well of a 6-well plate 12 h before transfection. Cells were transfected with pcDNA3.1-FLAG-GPΔMLD and increasing amounts of pCAGGS-3xHA-hMARCH8 expression vectors. After 48 h, cells were centrifuged at 2,000 rpm for 5 min and fixed with 4% paraformaldehyde for 10 min at RT. After brief centrifugation, cells were blocked with 5% bovine serum albumin and incubated with anti-FLAG primary antibody for 1 h at RT. After centrifugation and washing three times with PBS, Alexa Fluor 488-conjugated secondary antibody was added for 1 h at RT. After washing 3 times, cells were collected and analyzed by flow cytometry. To detect BiFC, 293T cells in 6-well plates were transfected with the pcDNA3.1-MARCH8-VN-HA/pcDNA3.1-GPΔMLD-FLAG-VC, pcDNA3.1-Furin-VN-HA/pcDNA3.1-GPΔMLD-FLAG-VC, or pcDNA3.1-MARCH8-VN-HA/pcDNA3.1-SERINC5-FLAG-VC pairs similarly and directly analyzed by flow cytometry.

### Western blotting.

Forty-eight hours posttransfection, cells were lysed with RIPA buffer (Sigma) and cytosolic fractions were collected. After incubation with loading buffer, samples were resolved by sodium dodecyl sulfate-polyacrylamide gel electrophoresis (SDS-PAGE). Some samples were subjected to Endo H or PNGase F treatment before applying them to the SDS-PAGE gel. For Endo H (catalogue no. P0702L; NEB) treatment, a total of 20 μg of cell lysate was first denatured by heating at 100°C for 10 min in denaturing buffer. Denatured proteins were added to a 20-μl reaction system containing 2 μl of GlycoBuffer and 1,000 units of Endo H and incubated at 37°C for 1 h. For PNGase F (catalogue no. P0708L; NEB) treatment, the same amount of cell lysate was added to 4 μl of PNGase F buffer (5×) to make a 20-μl total reaction volume and incubated at 80°C for 2 min. After cooling down,1 μl of Rapid PNGase F was added and samples were incubated 1 h at 37°C. After transferring to polyvinylidene difluoride (PVDF) membrane, 4% nonfat milk was used to block the membrane for 1 h at RT. The membrane was incubated with primary antibody and horseradish peroxidase (HRP)-conjugated secondary antibody. Alternatively, the membrane was incubated with HRP-conjugated primary antibody. Membranes were exposed after incubation with enhanced chemiluminescence (ECL) substrate (Thermo Fisher). Mouse anti-MARCH8 antibody was purchased from Proteintech; mouse anti-actin, -HA, and -FLAG monoclonal antibodies were purchased from Sigma; rabbit anti-EBOV GP and rabbit anti-IAV-HA polyclonal antibodies were purchased from Sino Biology (China). The mouse anti-HIV gp160, anti-HIV gp120, anti-HIV gp41, and anti-HIV p24/p55 monoclonal antibodies were obtained from the NIH AIDS Reagent Program. HRP-conjugated anti-human, anti-rabbit, or anti-mouse immunoglobulin G secondary antibodies were purchased from Pierce.

### Immunoprecipitation.

A total of 5 × 10^5^ 293T cells were seeded in each well of a 6-well plate 12 h before transfection. Forty-eight hours posttransfection, cells were lysed by RIPA buffer. Cytosolic fractions were collected and incubated with anti-FLAG antibody-conjugated magnetic beads (Sigma) overnight at 4°C. The beads were isolated by magnetic shelf, washed several times, and used for WB assay.

### Viral infectivity assay.

A total of 5 × 10^5^ 293T cells were seeded in each well of a 6-well plate 12 h before transfection. Cells were transfected with pNL-Luc-∆Env, pcDNA3.1-FLAG-GPΔMLD, and MARCH8 expression vectors. Forty-eight hours posttransfection, virion-containing supernatants were collected and subjected to ultracentrifugation at 40,000 × *g* for 1 h. Pellets were collected and analyzed by WB. In addition, 100-μl supernatants were collected for quantification by p24^Gag^ enzyme-linked immunosorbent assay (ELISA). Supernatants with equal amounts of p24^Gag^ were used to infect Vero E6 cells that were seeded in a 96-well plate. After another 48 h, infected cells were washed 3 times with PBS and lysed with 100 μl RIPA buffer. Twenty-five microliters was collected and mixed with an equal amount of substrate from the Bright-Glo luciferase assay system (Promega), and luminescence activity was determined by a luminometer.

## References

[1] Ng F, Tang BL 2016 Unconventional protein secretion in animal cells. Methods Mol Biol 1459:31–46. doi:10.1007/978-1-4939-3804-9_2. 27665549

[2] Viotti C 2016 ER to Golgi-dependent protein secretion: the conventional pathway. Methods Mol Biol 1459:3–29. doi:10.1007/978-1-4939-3804-9_1. 27665548

[3] Reily C, Stewart TJ, Renfrow MB, Novak J 2019 Glycosylation in health and disease. Nat Rev Nephrol 15:346–366. doi:10.1038/s41581-019-0129-4. 30858582 PMC6590709

[4] Frabutt DA, Zheng YH 2016 Arms race between enveloped viruses and the host ERAD machinery. Viruses 8:255. doi:10.3390/v8090255. PMC5035969 27657106

[5] Rey FA, Lok SM 2018 Common features of enveloped viruses and implications for immunogen design for next-generation vaccines. Cell 172:1319–1334. doi:10.1016/j.cell.2018.02.054. 29522750 PMC7112304

[6] York IA, Stevens J, Alymova IV 2019 Influenza virus N-linked glycosylation and innate immunity. Biosci Rep 39:BSR20171505. doi:10.1042/BSR20171505. 30552137 PMC6328934

[7] Stansell E, Panico M, Canis K, Pang PC, Bouche L, Binet D, O’Connor MJ, Chertova E, Bess J, Lifson JD, Haslam SM, Morris HR, Desrosiers RC, Dell A 2015 Gp120 on HIV-1 virions lacks O-linked carbohydrate. PLoS One 10:e0124784. doi:10.1371/journal.pone.0124784. 25915761 PMC4410959

[8] Cook JD, Lee JE 2013 The secret life of viral entry glycoproteins: moonlighting in immune evasion. PLoS Pathog 9:e1003258. doi:10.1371/journal.ppat.1003258. 23696729 PMC3656028

[9] White JM, Delos SE, Brecher M, Schornberg K 2008 Structures and mechanisms of viral membrane fusion proteins: multiple variations on a common theme. Crit Rev Biochem Mol Biol 43:189–219. doi:10.1080/10409230802058320. 18568847 PMC2649671

[10] Samji T, Hong S, Means RE 2014 The membrane associated RING-CH proteins: a family of E3 ligases with diverse roles through the cell. Int Sch Res Notices 2014:637295. doi:10.1155/2014/637295. 27419207 PMC4897099

[11] Lehner PJ, Hoer S, Dodd R, Duncan LM 2005 Downregulation of cell surface receptors by the K3 family of viral and cellular ubiquitin E3 ligases. Immunol Rev 207:112–125. doi:10.1111/j.0105-2896.2005.00314.x. 16181331

[12] Bartee E, Mansouri M, Hovey Nerenberg BT, Gouveia K, Fruh K 2004 Downregulation of major histocompatibility complex class I by human ubiquitin ligases related to viral immune evasion proteins. J Virol 78:1109–1120. doi:10.1128/jvi.78.3.1109-1120.2004. 14722266 PMC321412

[13] Goto E, Ishido S, Sato Y, Ohgimoto S, Ohgimoto K, Nagano-Fujii M, Hotta H 2003 c-MIR, a human E3 ubiquitin ligase, is a functional homolog of herpesvirus proteins MIR1 and MIR2 and has similar activity. J Biol Chem 278:14657–14668. doi:10.1074/jbc.M211285200. 12582153

[14] Bauer J, Bakke O, Morth JP 2017 Overview of the membrane-associated RING-CH (MARCH) E3 ligase family. N Biotechnol 38:7–15. doi:10.1016/j.nbt.2016.12.002. 27988304

[15] Tada T, Zhang Y, Koyama T, Tobiume M, Tsunetsugu-Yokota Y, Yamaoka S, Fujita H, Tokunaga K 2015 MARCH8 inhibits HIV-1 infection by reducing virion incorporation of envelope glycoproteins. Nat Med 21:1502–1507. doi:10.1038/nm.3956. 26523972

[16] Zhang Y, Tada T, Ozono S, Yao W, Tanaka M, Yamaoka S, Kishigami S, Fujita H, Tokunaga K 2019 Membrane-associated RING-CH (MARCH) 1 and 2 are MARCH family members that inhibit HIV-1 infection. J Biol Chem 294:3397–3405. doi:10.1074/jbc.AC118.005907. 30630952 PMC6416432

[17] Jeffers SA, Sanders DA, Sanchez A 2002 Covalent modifications of the Ebola virus glycoprotein. J Virol 76:12463–12472. doi:10.1128/jvi.76.24.12463-12472.2002. 12438572 PMC136726

[18] Wang B, Wang Y, Frabutt DA, Zhang X, Yao X, Hu D, Zhang Z, Liu C, Zheng S, Xiang SH, Zheng YH 2017 Mechanistic understanding of N-glycosylation in Ebola virus glycoprotein maturation and function. J Biol Chem 292:5860–5870. doi:10.1074/jbc.M116.768168. 28196864 PMC5392578

[19] Diehl WE, Lin AE, Grubaugh ND, Carvalho LM, Kim K, Kyawe PP, McCauley SM, Donnard E, Kucukural A, McDonel P, Schaffner SF, Garber M, Rambaut A, Andersen KG, Sabeti PC, Luban J 2016 Ebola virus glycoprotein with increased infectivity dominated the 2013–2016 epidemic. Cell 167:1088–1098.e6. doi:10.1016/j.cell.2016.10.014. 27814506 PMC5115602

[20] Urbanowicz RA, McClure CP, Sakuntabhai A, Sall AA, Kobinger G, Muller MA, Holmes EC, Rey FA, Simon-Loriere E, Ball JK 2016 Human adaptation of Ebola virus during the West African outbreak. Cell 167:1079–1087.e5. doi:10.1016/j.cell.2016.10.013. 27814505 PMC5101188

[21] Wool-Lewis RJ, Bates P 1998 Characterization of Ebola virus entry by using pseudotyped viruses: identification of receptor-deficient cell lines. J Virol 72:3155–3160. doi:10.1128/JVI.72.4.3155-3160.1998. 9525641 PMC109772

[22] Kerppola TK 2008 Bimolecular fluorescence complementation (BiFC) analysis as a probe of protein interactions in living cells. Annu Rev Biophys 37:465–487. doi:10.1146/annurev.biophys.37.032807.125842. 18573091 PMC2829326

[23] Dodd RB, Allen MD, Brown SE, Sanderson CM, Duncan LM, Lehner PJ, Bycroft M, Read RJ 2004 Solution structure of the Kaposi’s sarcoma-associated herpesvirus K3 N-terminal domain reveals a novel E2-binding C4HC3-type RING domain. J Biol Chem 279:53840–53847. doi:10.1074/jbc.M409662200. 15465811

[24] Thomas G 2002 Furin at the cutting edge: from protein traffic to embryogenesis and disease. Nat Rev Mol Cell Biol 3:753–766. doi:10.1038/nrm934. 12360192 PMC1964754

[25] Volchkov VE, Feldmann H, Volchkova VA, Klenk HD 1998 Processing of the Ebola virus glycoprotein by the proprotein convertase furin. Proc Natl Acad Sci U S A 95:5762–5767. doi:10.1073/pnas.95.10.5762. 9576958 PMC20453

[26] Wool-Lewis RJ, Bates P 1999 Endoproteolytic processing of the Ebola virus envelope glycoprotein: cleavage is not required for function. J Virol 73:1419–1426. doi:10.1128/JVI.73.2.1419-1426.1999. 9882347 PMC103966

[27] Dolnik O, Volchkova V, Garten W, Carbonnelle C, Becker S, Kahnt J, Stroher U, Klenk HD, Volchkov V 2004 Ectodomain shedding of the glycoprotein GP of Ebola virus. EMBO J 23:2175–2184. doi:10.1038/sj.emboj.7600219. 15103332 PMC424403

[28] Izaguirre G 2019 The proteolytic regulation of virus cell entry by furin and other proprotein convertases. Viruses 11:837. doi:10.3390/v11090837. PMC6784293 31505793

[29] Zhang Y, Tada T, Ozono S, Kishigami S, Fujita H, Tokunaga K 2020 MARCH8 inhibits viral infection by two different mechanisms. bioRxiv doi:10.1101/2020.04.09.035204. PMC7419139 32778221

[30] Braun E, Hotter D, Koepke L, Zech F, Groß R, Sparrer KMJ, Müller JA, Pfaller CK, Heusinger E, Wombacher R, Sutter K, Dittmer U, Winkler M, Simmons G, Jakobsen MR, Conzelmann K-K, Pöhlmann S, Münch J, Fackler OT, Kirchhoff F, Sauter D 2019 Guanylate-binding proteins 2 and 5 exert broad antiviral activity by inhibiting furin-mediated processing of viral envelope proteins. Cell Rep 27:2092–2104.e10. doi:10.1016/j.celrep.2019.04.063. 31091448

[31] Ahi YS, Yimer D, Shi G, Majdoul S, Rahman K, Rein A, Compton AA 2020 IFITM3 reduces retroviral envelope abundance and function and is counteracted by glycoGag. mBio 11:e03088-19. doi:10.1128/mBio.03088-19. 31964738 PMC6974572

[32] Li S, Ahmad I, Shi J, Wang B, Yu C, Zhang L, Zheng YH 2018 Murine leukemia virus glycosylated Gag reduces murine SERINC5 protein expression at steady-state levels via the endosome/lysosome pathway to counteract SERINC5 antiretroviral activity. J Virol 93:e01651-18. doi:10.1128/JVI.01651-18. PMC6321930 30355687

