# Supplementary material for: MARCH8 Inhibits Ebola Virus Glycoprotein, Human Immunodeficiency Virus Type 1 Envelope Glycoprotein, and Avian Influenza Virus H5N1 Hemagglutinin Maturation
Source: mBio. 2020 Sep 15;11(5):e01882-20. doi: 10.1128/mBio.01882-20 (PMC7492737; doi:10.1128/mBio.01882-20)
Supplement: FIG S1 [file mBio.01882-20-sf001.pdf]

Yu_Fig.S1

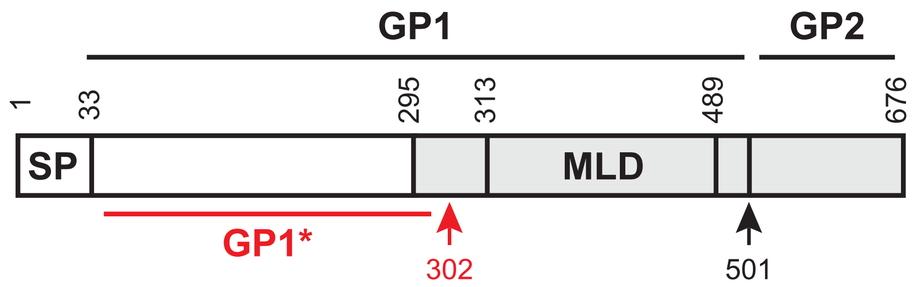

1 ... 33

MGVTGILQLPRDRFKRTSFFLWVIILFQRTFSIPLGVIHNSTLQVSDVDKLVCRDKLSSTNQLRS

VGLNLEGNGVATDVPSATKRWGFRSGVPPKVVNYEAGEWAENCYNLEIKKPDGSECLPAAPDGIR

GFPRCRYVHKVSGTGPCAGDFAFHKEGAFFLYDRLASTVIYRGTTFAEGVVAFLILPQAKKDFFS

SHPLREPVNATEDPSSGYYSTTIRYQATGFGTNETEYLFEVDNLTYVQLESRFTPQFLLQLNETI

299 302 313

YTSGKRSNTTGKLIWKVNPEIDTTIGEWAFWETKKNLTRKIRSEELSFTVVSNGAKNISGQSPAR

TSSDPGTNTTTEDHKIMASENSSAMVQVHSQGREAAVSHLTTLATISTSPQSLTTKPGPDNSTHN

TPVYKLDISEATQVEQHHRRTDNDSTASDTPSATTAAGPPKAENTNTSKSTDFLDPATTTSPQNH

489 501

SETAGNNNTHHQDTGEESASSGKLGLITNTIAGVAGLITGGRRTRREAIVNAQPKCNPNLHYWTT

QDEGAAIGLAWIPYFGPAAEGIYIEGLMHNQDGLICGLRQLANETTQALQLFLRATTELRTFSIL

NRKAIDFLLQRWGGTCHILGPDCCIEPHDWTKNITDKIDQIIHDFVDKTLPDQGDNDNWWTGWRQ

676

WIPAGIGVTGVIIAVIALFCICKFVF
